# The inhibitors of 17β-HSD10: are they any good?

**DOI:** 10.1039/d6cb00093b

**Published:** 2026-05-28

**Authors:** Ernst Kwa, Charlene E. Ogilvie, Natalie C. Kormos, Alison J. E. Green, Terry K. Smith, Frank J. Gunn-Moore

**Affiliations:** a School of Biology, University of St. Andrews St Andrews KY16 9ST UK fjg1@st-andrews.ac.uk; b Scottish Brain Sciences, Eden Campus, University of St. Andrews St Andrews Guardbridge KY16 0US UK; c School of Biology, University of St. Andrews St Andrews KY16 9ST UK

## Abstract

The advent of the first disease-modifying therapies for Alzheimer's disease (AD) has renewed optimism for effective prevention and treatment strategies. Growing mechanistic insights indicate that AD pathogenesis is multifactorial and non-linear, better conceptualized as a circular vortex in which interconnected pathological processes reinforce one another. This complexity highlights the necessity for multiple druggable targets and combination-based therapeutic approaches. A hallmark of AD is reduced cerebral glucose utilization, revealed by positron emission tomography studies, reflecting profound metabolic disruption and mitochondrial dysfunction. Among mitochondrial candidates, 17β-hydroxysteroid dehydrogenase type 10 (17β-HSD10), encoded by HSD17B10, has emerged as a protein of interest. Despite debate surrounding its substrate specificity due to conflicting *in vitro* data, its elevated expression in neurons and astrocytes within AD brains underscores its potential relevance. This review outlines chemical entities targeting both catalytic and non-catalytic functions of 17β-HSD10 and examines whether its inhibition offers biological efficacy and clarifies its metabolic roles in the living brain.

## Abstract 2

The advent of the first disease-modifying therapies for Alzheimer's disease (AD) has renewed optimism for effective prevention and treatment strategies. One particular strategy is to target the mitochondrial protein 17β-HSD10 as its activity has been linked to several aspects in AD pathogenesis.

## Graphical abstract

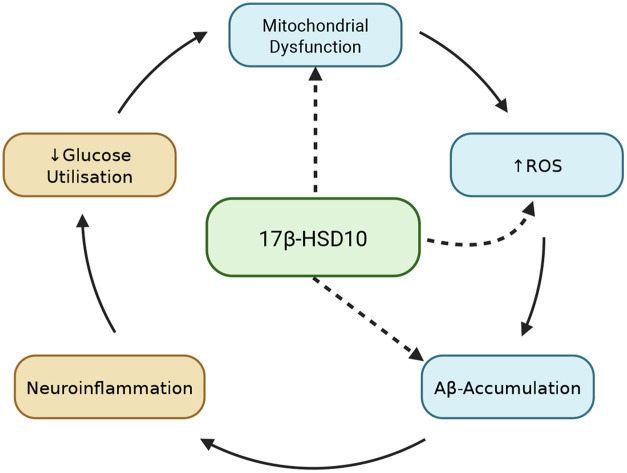

## Introduction

As the first disease-modifying treatments have started to appear,^1^ there is renewed optimism in the development of new therapeutics for the prevention and management of Alzheimer's disease (AD). Additionally, as research continues to shape our mechanistic understanding of AD pathology,^2,3^ it has become clear that disease triggers are multifactorial, and not simply a linear cascade of events. Consequently, we postulate that AD is a circular vortex with several entry points, and that pathogenesis rarely stems from a singular cause. Given the complex disease aetiology, identification of multiple druggable targets is imperative for effective therapeutics. Indeed, we have previously highlighted the requirement for at least three drug types to enable the effective treatment of AD.^4^

Previous studies using positron emission tomography (PET) scans have revealed a substantial reduction in glucose utilisation in AD brains, despite normal levels in healthy ageing counterparts.^5^ This finding indicates major disruptions in core metabolic processes, with mitochondrial dysfunction evidently being a well-documented feature of AD pathology.^6^ While 14 different mammalian classes/types of 17-β hydroxysteroid dehydrogenase (17β-HSD) have been identified, only 12 are present in humans^7,8^

The focus of this review is 17-β hydroxysteroid dehydrogenase type 10 (17β-HSD10), encoded by the HSD17B10 gene.^9–11^ This mitochondrial enzyme has piqued longstanding interest and generated much debate, with several publications disputing proper nomenclature due to contradictory *in vitro* findings, leading to uncertainty over substrate specificity *in vivo*.^12–15^ Such discrepancies, however, can be attributed to wide variations in experimental parameters, which likely have little relevance to the living human brain. What is not in question, however, is that both neurons and astrocytes are involved, with each reported to have elevated 17β-HSD10 protein expression in the AD brain,^12,16^ although the full consequence of this has yet to be elucidated.

In the last decade, several different lines of research have suggested that modulation of 17β-HSD10 function may have therapeutic merit. Therefore, in this review, we will describe the different chemical entities that have been developed to target the catalytic and non-catalytic activities of this protein. Many of these novel compounds are now at the stage of being able to answer two critical questions:

(1) Does inhibiting 17β-HSD10 activity have biological efficacy in AD models (and possibly other diseases)?

(2) Is it now possible to determine which metabolites/substrates 17β-HSD10 modifies in the living brain?

## 17β-HSD10 identification and nomenclature

As outlined above, identification of the true physiological role of 17β-HSD10 has been a longstanding point of contention in the field, largely due to early studies relying upon heterologous expression of recombinant proteins in *Escherichia coli*, with much variation in subsequent protein purification and activity assay protocols.^12,14,17–19^ However, a combination of *in vivo* and cell culture studies in mammals, *Xenopus laevis* and *Drosophila melanogaster* has given some insights into physiologically relevant functions, highlighting clear roles in neurosteroid oxidation, mitochondrial homeostasis and branched-chain amino acid metabolism.^12,19,20^ For example, deletion of the 17β-HSD10 gene in mouse models resulted in embryonic demise during gastrulation,^21^ while a tissue-specific conditional knockout led to the development of mitochondrial abnormalities, such as the loss of cristae and fragmented organelle.^21^ In *X. laevis* models, knockdown of the 17β-HSD10 homologue reduced mitochondrial pyruvate turnover and triggered apoptosis that eliminated forebrain and eye structures.^21^ Conversely, rescue experiments demonstrated that microinjection of wild-type human 17β-HSD10 restored mitochondrial morphology and prevented apoptosis.^21^ Furthermore, loss-of-function studies investigating the *D. melanogaster* short chain l-3-hydrxoyacyl-CoA dehydrogenase, a 17β-HSD10 homologue encoded by the *scully* (*scu*) gene, revealed embryonic and pupal lethality, accompanied by severe defects in germline development; mutant flies also exhibited mitochondrial abnormalities in photoreceptor cells, producing phenotypes that closely resembled human β-oxidation fatty acid disorder.^22^ Overexpression studies have further illustrated the functional significance of 17β-HSD10.^23^ For example, overexpression of 17β-HSD10 in PC12, rat adrenal gland tumour-derived cells, accelerated tumour growth in both culture monolayers and in severe combined immunodeficiency (SCID) mouse xenografts; this enhanced tumorigenicity was accompanied by increased mitochondrial complex IV activity and upregulated ATP production.^23^

The broad substrate range of 17β-HSD10 may explain why this enzyme was independently “rediscovered” several times across different fields, leading to a series of nomenclatures that reflect its diverse biological roles. For example, one of the earliest identifications of 17β-HSD10 in mammalian systems came from a yeast two-hybrid screen in HeLa cells. Here, it was determined to bind the amyloid beta (Aβ) peptide and was reported to localise to the endoplasmic reticulum (ER); hence, it was named endoplasmic reticulum amyloid binding (ERAB) protein.^12^ Almost simultaneously, biochemical purification studies^14^ identified a mitochondrial short-chain dehydrogenase (SCHAD) and classified it as l-3-hydroxyacyl-CoA dehydrogenase based upon its activity towards β-oxidation intermediates.^14,18^ Shortly thereafter, the same gene product was proposed to possess alcohol dehydrogenase activity and ability to bind Aβ directly, giving rise to the revised name Aβ-binding alcohol dehydrogenase (ABAD).^17^ This naming was also a departure from the previously used ERAB, and the associated misconception of sole localization in the endoplasmic reticulum, which was overturned after new discoveries, obtained through GFP-tagged intracellular experiments, evidently localized the protein within the mitochondria.^24^ In parallel, clinical geneticists investigating inborn errors of metabolism identified a male patient with X-linked neurodegenerative disease caused by mutations in the same gene.^25^ The authors coined the protein product 2-methyl-3-hydroxybutyryl-CoA dehydrogenase (MHBD)^15,25^ and proposed a crucial role in isoleucine catabolism. It was recognised only later that these independently described proteins were identical entities. Previously, in 1996, the enzyme was identified in bovine mitochondria,^26^ and mass spectrometry work of Ofman *et al.* (2003)^15^ later revealed that purified MHBD from bovine liver was identical to purified bovine 3-hydroxyacyl-CoA dehydrogenase type II (HADH2), resulting in the combining of science under these two names. The name HADH2 was attributed to the discovered function of catalysing the redox conversion between l-3-hydroxyacyl-CoA and 3-ketoacyl-CoA.^14^ Ofman *et al.* (2003)^15^ recognized the human homolog of this studied bovine enzyme as being ERAB within the short-chain 3-hydroxyacyl-CoA dehydrogenase family. The unifying nomenclature, 17β-HSD10, was subsequently adopted following the discovery that the enzyme also catalyses the oxidation of 17β-estradiol (E2), allopregnanolone (AlloP) and 3α-androstanediol.^27^ In 2008, the focal enzyme was also termed MRPP2 by Holzmann *et al.*, named for its association with mtRNase P activity, but was clearly also identified by its accompanying breadth of names.^28^ Although it has also been referred to as SDR5C1, denoted for its membership in the short-chain dehydrogenase/reductase superfamily,^29–31^ the focal enzyme of this review is presently identified as 17β-HSD10^32^. In 2007, the gene symbol, HSD17B10, was officially adopted by the Human Gene Nomenclature Committee (HGNC)^33^ and has since been used consistently across multiple studies.

## Endogenous 17β-HSD10 substrates and functions

### 1 Steroidal substrates

**Fig. 1:**
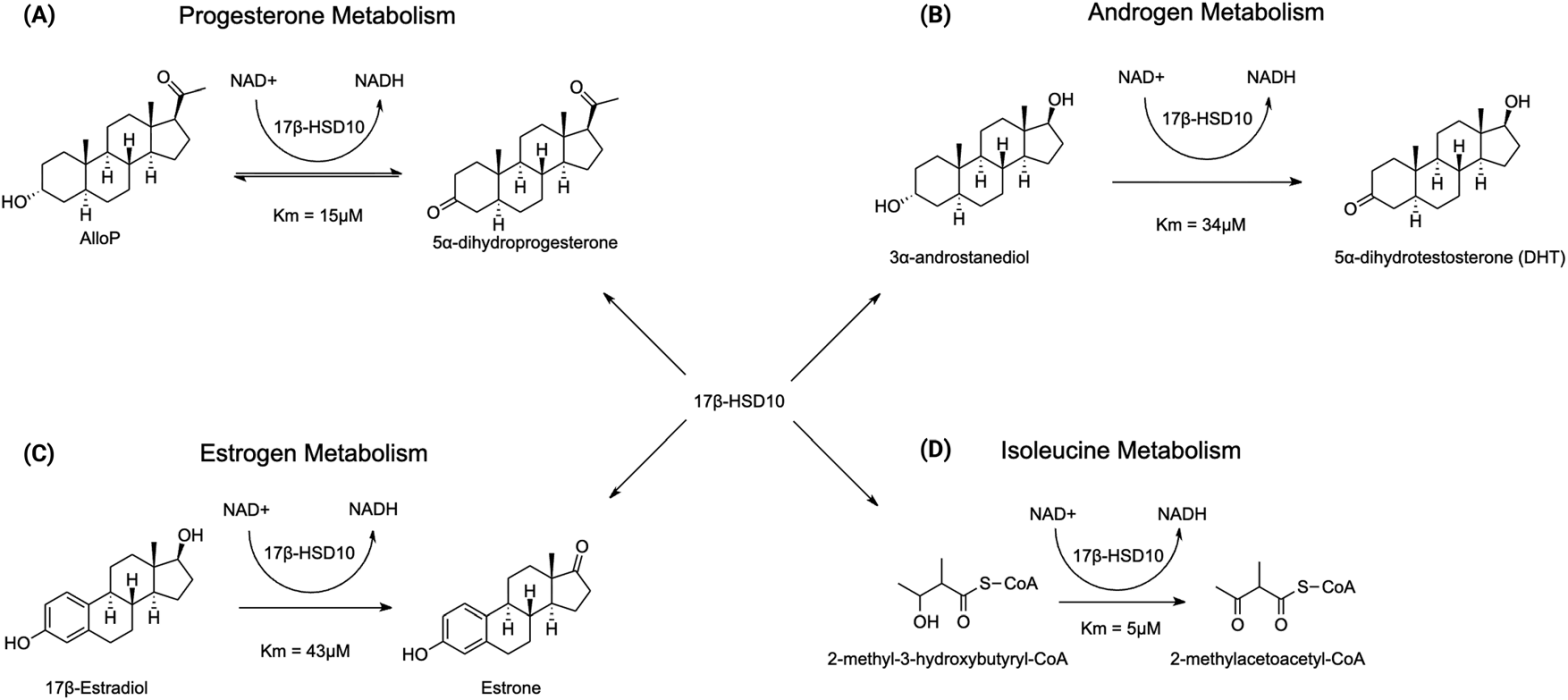
Reactions catalysed by 17β-HSD10 in steroid and isoleucine metabolism. (A) In progesterone metabolism, AlloP is oxidised to form 5α-dihydroprogesterone. (B) In androgen metabolism, 3α-androstanediol is converted to 5α-dihydrotestosterone (DHT). (C) In estrogen metabolism, 17β-estradiol is oxidised to estrone. (D) In isoleucine catabolism, 2M3HBA is converted to 2MAA-CoA. In all reactions, NAD^+^ serves as a cofactor and is reduced to NADH. *K*_m_ values are reported in ref. 19, 34 and 35.

The lipid-soluble steroid hormones, oestrogens and androgens, are amongst the most important physiological substrates of 17β-HSD10.^34^ These cholesterol-derived signalling molecules play vital roles in a wide range of processes, including reproduction, development, metabolism and immune function.^35–38^ In particular, neurosteroids are known to be crucial in brain development, neuroprotection and neurogenesis, with dysregulation strongly implicated in both HSD10 deficiency and AD.^39^ For example, AlloP, which is oxidised by 17β-HSD10 to form 5α-dihydroprogesterone (5α-DHP) in an NAD^+^-dependent manner (Fig. 1A), is a potent positive allosteric modulator of γ-aminobutyric acid type A receptors (GABA_A_R) and is essential for maintaining a variety of neurological functions, including normal GABAergic tone.^40^ Investigations have shown that excessive 17β-HSD10 activity disrupts GABAergic signalling, which ultimately contributes to excitotoxic vulnerability *via* reduction in AlloP bioavailability.^19,40^

Furthermore, 17β-HSD10 also catalyses the oxidation of 3α-androstanediol into dihydrotestosterone (DHT) (Fig. 1B), linking the enzyme to androgen metabolism.^19^ In prostate cancer, this reaction contributes to a non-classical androgen synthesis pathway, allowing tumour cells to generate DHT even under androgen-deprivation therapy.^41,42^ This bypass mechanism provides an additional source of DHT, despite suppressed circulating testosterone, and promotes continued androgen receptor signalling, often giving rise to treatment resistance.^43^

One of the most significant reactions catalysed by 17β-HSD10 is the oxidation of E2 (estradiol) to E1 (estrone) (Fig. 1C), with the former supporting essential roles in both female and male physiology, contributing to reproductive function, lipoprotein synthesis, prevention of genital atrophy, regulation of insulin sensitivity and maintenance of cognitive and neuronal function.^44,45^ Beyond its canonical role as a sex hormone, E2 acts directly on neurons, astrocytes, microglia and neural stem cells, where it modulates ion channel activity and intracellular signalling cascades such as the cyclic adenosine monophosphate (cAMP),^46^ mitogen-activated protein kinase (MAPK)^47^ and protein kinase B (Akt)^48^ pathways. Additionally, upon binding to nuclear estrogen receptors alpha (ERα) and beta (ERβ), E2 induces receptor dimerisation and translocation to the nucleus, regulating gene expression in a cell-specific manner,^49^ and subsequently influencing cognition, mood, motor function and neuroprotection.^50–52^

Critically, a substantial portion of E2's neuroprotective effects are mediated *via* signalling pathways that converge on cellular survival mechanisms, particularly within the mitochondria.^53,54^ In primary hippocampal neurons, E2 activates the phosphatidylinositol 3-kinase (PI3K/Akt) and MAPK signalling cascades to protect against glutamate-induced cytotoxicity.^55,56^ These pathways stabilise mitochondrial calcium buffering and increase the expression of anti-apoptotic proteins, such as B cell lymphoma-2 (BCl-2), thus preventing initiation of the apoptotic cascade.^53^ In addition, E2 also plays a crucial role in neuronal bioenergetics, enhancing mitochondrial efficiency by upregulating key metabolic enzymes such as pyruvate dehydrogenase, complex IV of the electron transport chain (ETC) and ATP synthase, thereby expediting glycolysis and oxidative phosphorylation.^53,57^ Consequently, E2 serves to reduce mitochondrial oxidative stress, preserve membrane potential and maintain overall mitochondrial integrity.^58^ At the synaptic level, E2 rapidly enhances dendritic spine formation through ERβ activation in cortical neurons.^59^ This facilitates synaptic transmission and long-term potentiation (LTP) in the hippocampus by activating the RhoA/ROCK signalling pathway, facilitating actin polymerisation and stabilisation of the synaptic cytoskeleton.^59,60^

Collectively, these mechanisms highlight E2 as a critical neuroprotective steroid whose actions span mitochondrial regulation, synaptic plasticity and pro-survival signalling. Consequently, aberrant 17β-HSD10 activity, be it through overexpression^23^ or Aβ-mediated dysregulation,^12^ has the potential to disrupt E2 signalling and therefore contribute to mitochondrial dysfunction, synaptic instability and increased vulnerability to neurodegenerative diseases such as AD. Moreover, the decline in circulating estradiol levels during menopause may further exacerbate these effects, leading to a decrease in E2-mediated neuroprotective effects and increasing susceptibility to AD in menopausal women.^61^

### 2 Non-steroidal substrates

**Fig. 2:**
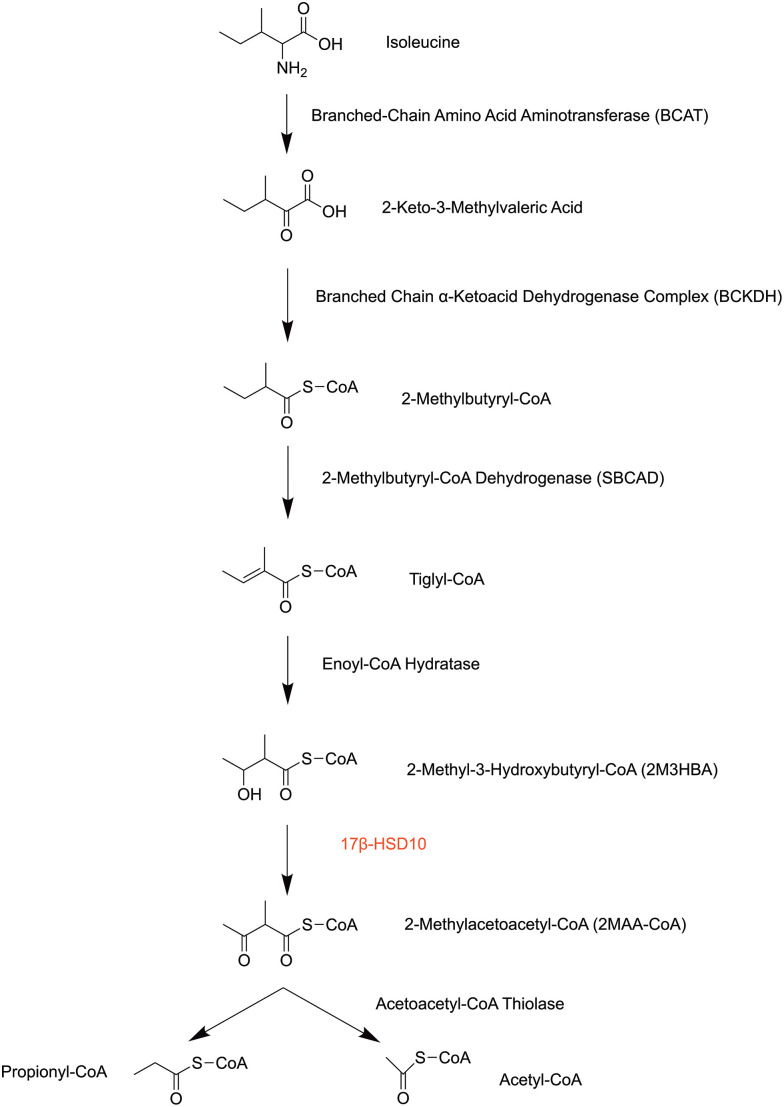
Schematic overview of the mitochondrial degradation of isoleucine to acetyl-CoA and propionyl-CoA. Isoleucine is first transaminated to 2-keto-3-methylvaleric acid by branched-chain amino acid aminotransferase (BCAT), followed by oxidative decarboxylation by the branched-chain α-ketoacid dehydrogenase complex (BCKDH) to yield 2-methylbutyryl-CoA. Sequential enzymatic steps catalysed by 2-methylbutyryl-CoA dehydrogenase (SBCAD) and enoyl-CoA hydratase produce tiglyl-CoA and 2M3HBA, respectively. 17β-HSD10 then catalyses the NAD^+^-dependent oxidation of 2M3HBA to 2MAA-CoA which is subsequently cleaved by acetoacetyl-CoA thiolase to generate acetyl-CoA and propionyl-CoA.^63,70^

In addition to its roles in neurosteroid and systemic steroid metabolism, 17β-HSD10 also mediates catabolism of branched chain amino acids (BCAAs) within the mitochondria, catalysing a key step in the isoleucine degradation pathway (Fig. 1D) *via* oxidation of 2-methyl-3-hydroxybutyryl-CoA (2M3HBA) to 2-methylacetoacetyl-CoA (2MAA-CoA) in an NAD^+^-dependent manner.^25,62^ Unlike the other BCCAs, leucine and valine, isoleucine is both ketogenic and glucogenic, ultimately giving rise to acetyl-CoA and propionyl-CoA (Fig. 2), respectively.^63^ The former is a major precursor in the synthesis of ketone bodies and fatty acids,^64^ while the latter contributes to gluconeogenesis *via* conversion to oxaloacetate,^65,66^ with both acting as key intermediates in the tricarboxylic acid cycle (TCA), generating high-energy electron carriers NADH/FADH_2_ for oxidative phosphorylation and GTP/ATP by substrate-level phosphorylation.^15,67,68^ Evidently, the importance of isoleucine metabolism is further supported by genome-wide association studies (GWAS), which identified a positive relationship between genetic predisposition to raised plasma isoleucine levels and the development of AD.^69^ This critical factor reinforces the importance of proper 17β-HSD10 activity to sustain mitochondrial energy supply, particularly in high-demand tissues such as the brain.

As briefly alluded to above, HSD10 deficiency, also referred to as HSD10 disease, 17β-HSD10 deficiency, HSD10 mitochondrial disease and MHBD deficiency, is a rare X-chromosome-linked disease caused by missense mutations of the HSD17β10 gene.^71–74^ First identified in 2000, in a 2-year-old male patient through urinary metabolite analysis, this rare disease is characterized by clinical symptoms of neurodegeneration, impaired motor control, psychomotor delay, regression of previously acquired motor and cognitive skills, choreoathetosis, cardiomyopathy, abnormal metabolic function, seizures,^25,73^ cognitive impairment, epilepsy,^10^ retinopathy^74^ and mitochondrial dysfunction.^75^ The clinical phenotype is dependent upon the exact mutation position.^32^ Clinical forms of this disease present as neonatal, infantile and juvenile forms, all with severely reduced patient lifespan.^10^ It was subsequently determined that loss of enzymatic function results in the upstream accumulation of 2M3HBA, producing a toxic organic acid load that can be detected in urine as 2-methyl-3-hydroxybutyrate (2M3HB).^15,25^ This metabolic block prevents the downstream formation of acetyl-CoA and propionyl-CoA, substantially impairing mitochondrial energy production.^15^ There is currently no known effective treatment,^10,73,74^ and while adopting an isoleucine-restricted diet may decrease the metabolites (2M3HB and tiglyglycine) in the urine, clinical symptoms of this deficiency do not exhibit any improvement or slow in progression.^21,25,73,74,76^ While it was initially thought that HSD10 deficiency pathology arose exclusively due to inborn errors in isoleucine metabolism,^25^ this earlier hypothesis was proved incorrect following the discovery in human tissues that mitochondrial energy failure in HSD10 deficiency is caused by abnormal mitochondrial RNA processing.^77^ This came after 17β-HSD10 was identified to be a component of the mitochondrial RNase P complex,^28^ which provides post-transcriptional processing of RNAs into mitochondrial mRNAs, tRNAs and rRNAs.^77^ Northern blots performed on heart tissue from control and diseased patients revealed elevated levels of unprocessed RNA in samples with HSD10 deficiency.^77^ The disease phenotypes ultimately arise from failure in mitochondrial energy production within the developing nervous system. Taken together, these findings demonstrate that impaired isoleucine catabolism and irregular mitochondrial RNA processing lead to augmented metabolic stress and serve as key contributors to neuronal death.

Similarly, decreased 17β-HSD10 levels have been measured in the brains of Parkinson's disease (PD) patients.^72,78^ PD is a neurodegenerative disease with a primary neuropathological hallmark of degradation of the dopaminergic neurons in the substantia nigra pars compacta in the ventral midbrain, a region essential for movement and motor control.^72,78^ PD is characterized by primary impairments of rest tremor, bradykinesia, limb and trunk stiffness and postural instability with freezing of gait.^79^ Compared to wild-type littermates, transgenic mice overexpressing human 17β-HSD10 fourfold were shown to be more resistant to the neurotoxin 1-methyl-4-phenyl-1,2,3,6-tetrahydropyridine, a toxin which impairs mitochondrial respiration and degrades dopaminergic neurons.^78^ In this same series of experiments, western blot analysis of human tissue samples obtained from the Parkinson Brain Bank at Columbia University revealed reduced levels of 17β-HSD10 in the ventral midbrain region of post-mortem samples of PD patients when compared to control subjects.^78^ Thus, there is evidently a required balance of the expression of this enzyme,^80^ in prevention of AD at one end and PD at the other.^23^ Further investigation could provide treatment clarity as to the more specific importance of the effects of altering the expression of a catalytically inactive vs active form of the enzyme and subsequently provide a therapeutic foundation for treatment of PD and HSD10 deficiency.

### 3 Non-enzymatic functions of 17β-HSD10

While enzymatic activities in steroid and isoleucine metabolism are well-established, 17β-HSD10 has also been shown to operate *via* noncatalytic interactions with other proteins, including ERα, cyclophilin D (CypD) and the mitochondrial RNase (mtRNase) P complex.^23,28,81,82^

**Fig. 3:**
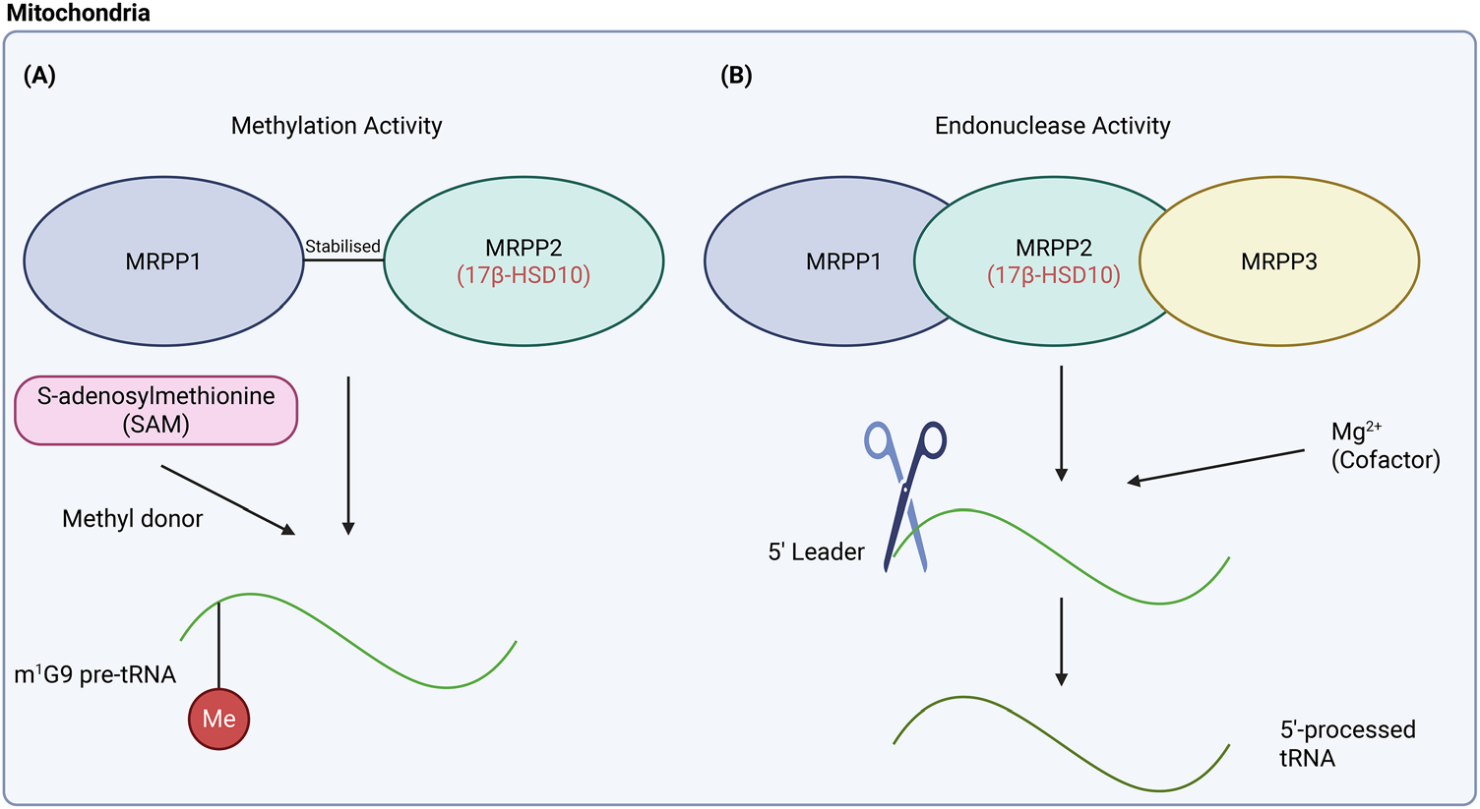
Non-enzymatic role of 17β-HSD10 (MRPP2) in mitochondrial tRNA processing. 17β-HSD10 forms a complex with MRPP1 and MRPP3 in mitochondria to regulate tRNA maturation. (A) In the methylation pathway, MRPP1-17β-HSD10 is associated with catalyse *N*^1^-methylation of guanosine at position 9 (m^1^G9) of precursor tRNA using *S*-adenosylmethionine (SAM) as a methyl donor,^88^ with 17β-HSD10 providing a structure stabilising role.^28^ (B) In the endonuclease pathway, the MRPP1-17β-HSD10-MRPP3 complex forms mtRNase P,^28^ where MRPP3 functions as the catalytic endonuclease, responsible for 5′ cleavage of precursor tRNA.^28^ This cleavage requires Mg^2+^ as a cofactor and generates mature 5′-processed tRNA.^28^ Created in BioRender. Lab, G. (2026) https://BioRender.com/fw89upy.

Specifically, 17β-HSD10 forms a crucial component of the human mitochondrial RNase P (mtRNase P) complex.^28^ This multi-subunit complex serves as a protein-only endonuclease that cleaves the 5′ leader sequence from pre-transfer tRNA (tRNA) to generate mature tRNA^83^ (Fig. 3). Human mtRNase P was long presumed to contain a trans-acting RNA component, analogous to the ribozyme-based RNase P systems found in prokaryotes and eukaryotic nuclei.^84^ This assumption, however, was disproven when the mitochondrial enzyme was found to be composed entirely of three nuclear-encoded protein subunits: tRNA methyltransferase (MRPP1/TRMT10C), 17β-HSD10 (MRPP2/SDR5C1) and Mg^2+^-dependent endoribonuclease (MRPP3/PRORP).^28,85^ Within this complex, MRPP2 forms a stable subcomplex with MRPP1, serving as a structural platform, with ancillary roles in mitochondrial tRNA-binding and methylation, which facilitate catalytic activity of the MRPP3 subunit.^28^ The MRPP2, *i.e.* the 17β-HSD10 component, is comprised of a homotetramer, with each monomer adopting a Rossmann fold to yield a dehydrogenase active site capable of binding the NAD^+^ cofactor;^28^ however, within the context of mtRNase P, this domain is repurposed as a tRNA-binding motif.^28,86^ The importance of MRPP2 has been highlighted by knockdown studies, which demonstrated that MRPP2 knockdown resulted in the accumulation of unprocessed mitochondrial tRNA precursors and impaired mitochondrial translation machinery.^28,77^ Furthermore, MRPP2 has also been identified as a critical determinant for the stable expression of MRPP1, experimentally evidenced, for example, where single point mutations, such as R130C, in 17β-HSD10, led to a reduction in MRPP2 and a concomitant loss of MRPP1, resulting in impaired RNase P activity and defective RNA processing.^87^

**Fig. 4:**
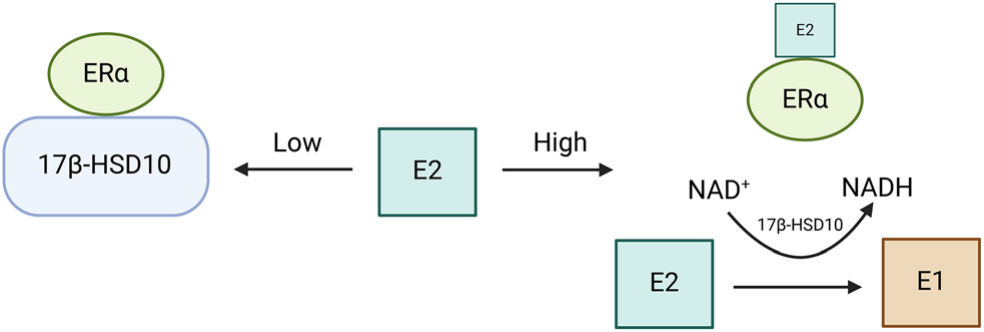
A diagram depicting the interaction of ERα and 17β-HSD10. Under low concentrations of E2, 17β-HSD10's enzymatic activity is inhibited by ERα. In the presence of high E2, ERα dissociates from 17β-HSD10, allowing 17β-HSD10 to catalyse the conversion of highly potent E2 to less potent E1. Figure adapted from ref. 81, *Biochemical and Biophysical Research Communications*, **384**(4), V. Jazbutyte, F. Kehl, L. Neyses and T. Pelzer, Estrogen receptor alpha interacts with 17β-hydroxysteroid dehydrogenase type 10 in mitochondria, pp. 450–454, copyright 2009, with permission from Elsevier. Created in BioRender. Lab, G. (2026) https://BioRender.com/on5sshe.

Another non-enzymatic interaction between 17β-HSD10 and ERα (Fig. 4) within the mitochondria has been documented, suggesting that the enzyme plays a role in regulating local hormone signalling. This interaction was first observed in neonatal rat cardiomyocytes, where 17β-HSD10 was found to bind directly to the ligand-binding domain of ERα in a hormone-sensitive manner.^81^ In these studies, the complex remained intact and 17β-HSD10 activity was inhibited under low estrogen conditions; however, when mitochondrial E2 levels increased, ERα dissociated from the complex, allowing 17β-HSD10 to freely oxidise E2 to E1, ultimately resulting in signal termination. This characteristic led to a model in which 17β-HSD10 functions as a mitochondrial estrogen sensor and regulator.^81^

In addition, another important binding partner of 17β-HSD10 is the mitochondrial regulator CypD. A combination of co-immunoprecipitation and immunofluorescence studies in PC12 cells has shown that overexpression of 17β-HSD10 increased the formation of the 17β-HSD10-CypD complex, whereas knockdown of 17β-HSD10 yielded a reduction in CypD levels.^23^ Under oxidative stress, CypD typically translocates from the mitochondrial matrix into the inner mitochondrial membrane, where it facilitates opening of the mitochondrial permeability transition pore (MPTP), a key event that drives apoptosis or necrosis.^23,89^ Enhanced binding of CypD by 17β-HSD10 appears to retain CypD within the matrix, thus preventing stress-induced translocation. As such, MPTP opening is inhibited, conferring resistance to oxidative stress-induced mitochondrial dysfunction and limiting misguided apoptosis that would conversely lead to cell death. This interaction suggests that 17β-HSD10 can modulate mitochondrial vulnerability to injury, in addition to its enzymatic activities.

## Dual mechanisms of 17β-HSD10 in Alzheimer's disease pathogenesis

The mitochondrial enzyme 17β-HSD10 additionally plays a critical role in the pathophysiology of AD through two primary mechanisms, both of which contribute to neurodegeneration.

The first pathological mechanism is the overexpression of 17β-HSD10 in AD brains, which, as described above, disrupts neurosteroid metabolism and homeostasis.^71^ Excessive enzymatic activity accelerates the degradation of E2 and AlloP, leading to decreased levels of these important protective neurosteroids.^90,91^ This reduction leads to decreased ATP production, causing dysregulation of bioenergetics and redox homeostasis, which contributes to mitochondrial dysfunction.^92,93^ Independent of AD, cellular studies have shown that overexpression of 17β-HSD10 can itself induce cellular damage, reduce viability and impair mitochondrial function in a manner dependent upon its catalytic activity, suggesting that it acts as an independent pathological factor.^71,94^

The second major pathological role of 17β-HSD10, involves its interaction with one of the well-documented pathogenic hallmarks of AD, Aβ aggregates.^93^ The most direct link is 17β-HSD10's ability to bind Aβ, particularly the plaque-forming isoforms Aβ(1–40) and Aβ(1–42).^20^ Aβ enters neuronal mitochondria, where it associates with 17β-HSD10 *via* a unique binding site called loop D (92–120); this region forms a short β-hairpin structure exposing key residues, Thr108, His109 and Thr110 for protein–protein interactions.^20,95^ It is thought that binding of Aβ to 17β-HSD10 results in a conformational change, which prevents normal enzymatic function *via* blockage of the NAD^+^ cofactor and/or substrate binding sites.^20^ This binding event has been shown to be cytotoxic *in vitro*, leading to an increase in reactive oxygen species (ROS), inhibition of mitochondrial complex IV and the release of cytochrome *c* and lactate dehydrogenase, ultimately leading to apoptosis.^20,95,96^ Evidence from cell viability assays and transgenic experiments shows that disruption of the Aβ-17β-HSD10 interaction leads to positive effects on mitochondria, mitigating oxidative stress and Aβ-induced toxicity.^20^

## Inhibitors of 17β-HSD10

Following the above, the targeted modulation of 17β-HSD10 presents a promising direction for the development of future therapeutics for the treatment of AD.^43^ The goal is to mitigate Aβ-induced cytotoxicity, while restoring neuroprotective steroidal balance in neurons. Unfortunately, the search for novel and promising inhibitors of 17B-HDS10 has proved challenging. To date, several compounds have been repurposed or created *de novo* to target either Aβ-17β-HSD10 interactions or to directly inhibit 17β-HSD10 activity.^12,20,97^ Based upon their structure and function, these inhibitors can be broadly classified into 5 major groups: loop D mimetics, benzothiazolyl ureas, fused pyrazole compounds, steroidal compounds, and repurposed drugs and other compounds.

## Loop D mimetics

**Fig. 5:**
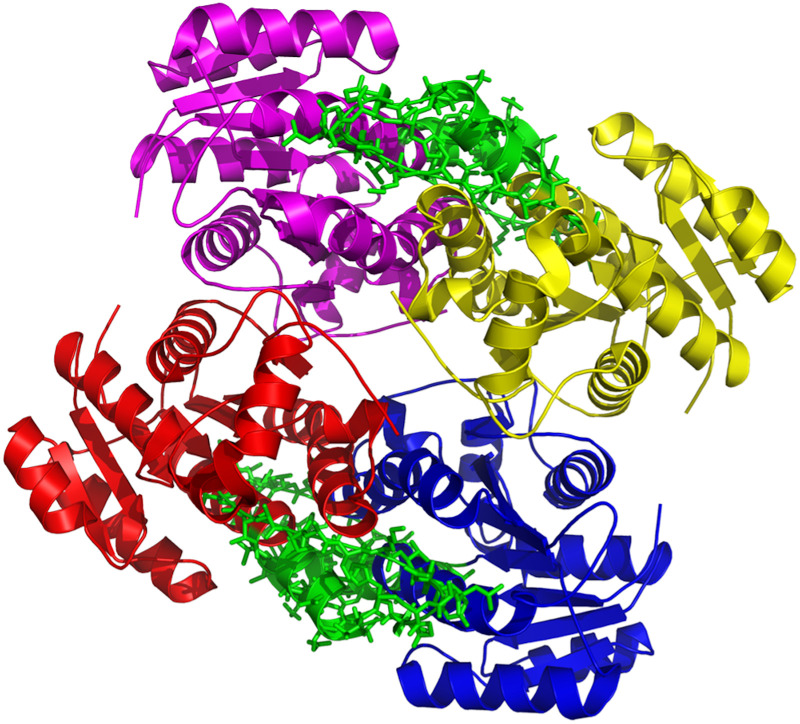
Crystal structure of the human 17β-HSD10 homotetramer with each subunit represented by different colours and the loop D region highlighted in green (PDB: 1U7T).

Early efforts to disrupt the pathological interaction between 17β-HSD10 and Aβ focused on peptide-based mimetics derived from the loop D region of the enzyme, which forms the Aβ-binding interface (Fig. 5). For example, Lustbader *et al.* (2004)^20^ first synthesised a peptide dubbed ABAD-decoy peptide (ABAD-DP) using the corresponding amino acid residues (92–120). The peptide effectively inhibited binding between 17β-HSD10 and both Aβ(1–40) and Aβ(1–42) *in vitro*, with IC_50_ values of 4.9 µM and 1.7 µM, respectively. To investigate peptide activity *in vivo*, ABAD-DP was bioengineered to incorporate additional sequences.^98^ The construct encoded the Tat protein transduction domain of the HIV1 virus to ensure transport across the cell membrane and blood–brain barrier (BBB) and a mitochondrial targeting sequence (Mito) to ensure specificity translocation into neuronal mitochondria. This construct, termed Tat-Mito-DP, effectively inhibited the Aβ–17β-HSD10 interaction, which preserved mitochondrial function and improved spatial memory in transgenic mouse models of AD; however, the therapeutic potential of this peptide was limited by its short *in vivo* half-life.^98^ To address the issue, an attempt at stabilisation was ventured *via* fusion with thioredoxin-1 (Trx-1).^99^ This modification enhanced stability and prolonged ABAD-DP activity, allowing the conjugate to successfully protect against Aβ-induced cytotoxicity and re-establish redox balance in PC12 cells.^99^ However, later studies using the thioredoxin-fused peptide aptamers shifted focus towards disrupting interactions between toxic Aβ assemblies and cellular prion proteins (PrP^c^).^100^

Given the limited bioavailability and short half-life of peptide-based mimetics, efforts were refocused on developing non-peptidyl small molecule inhibitors to target the loop D interface.^95^ A combination of computational and structural analyses identified three key residues: Thr108, His109 and Thr110, which act as hotspots mediating Aβ–17β-HSD10 interactions.^95^ Virtual screening of chemical databases formed the basis of *in silico* drug discovery, followed by enzyme-linked immunosorbent assay (ELISA)-based verification of the top 20 hits, which identified 2 lead compounds, VC15 and VC19 (Table 1), with IC_50_ values of 4.4 µM and 9.6 µM, respectively.^95^ At a structural level, the inhibitory activity of VC15 and VC19 is mediated by hydrophobic interactions and hydrogen bonding that engages with Aβ residues such as Phe4, Glu11 and Gln15 within the loop D pocket. The authors noted that of the two compounds, VC19 yielded a superior binding score. VC19, however, demonstrated lower *in vitro* potency compared to VC15. This difference in potency was ascribed to VC15 forming a greater number of polar interactions within the Aβ-binding pocket compared to VC19.^95^

**Table 1:** Names and structures of loop D mimetic inhibitors. Inhibitor names are shown as they appear in the original literature

Compound	IC_50_ (µM)	Assay type	Inhibition type	Type	Sequence/structure	Ref.
ABAD-DP	1.70	Cell based	Aβ-interaction	Peptide	AGIAVASKTYNLKKGQTHTLEDFQRVLDV	20
Tat-Mito-ABAD-93-116	—	Mice based	Aβ-interaction	Peptide	YGRKKRRQRRR-MAAAVRSVKGL-GIAVASKTYNLKKGQTHTLEDFQR	98
VC15	4.40	ELISA based	Aβ-interaction	Small molecule	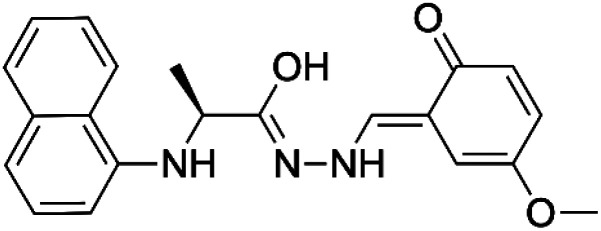	95
VC19	9.60	ELISA based	Aβ-interaction	Small molecule	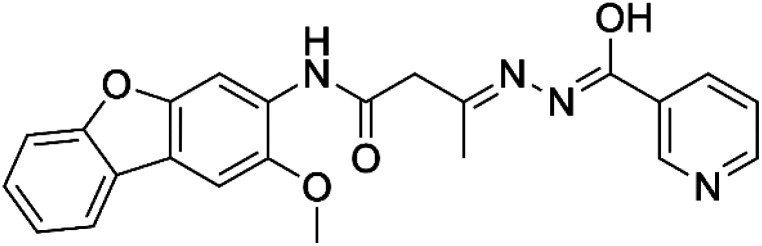	95

## Benzothiazolyl ureas

Benzothiazolyl ureas are a class of organic compounds consisting of a benzothiazolyl heterocycle linked *via* a urea bridge to various aryl moieties.^101,102^ These small molecules display diverse biological activities, and their scaffolds are routinely utilised in medicinal chemistry, forming the basis of various anti-cancer, anti-bacterial and AD therapeutics.^103^ The discovery of benzothiazolyl ureas as inhibitors of 17β-HSD10, originated from ELISA-based screening assays designed to identify 17β-HSD10 and Aβ binding partners.^104^ These investigations identified frentizole, an FDA-approved immunosuppressive drug, as a novel 17β-HSD10–Aβ inhibitor, with an IC_50_ of 200 µM (Table 2).^104^ Despite initially exhibiting suboptimal potency, the frentizole scaffold provided an important structural template for further optimisation, leading to the synthesis of a series of benzothiazolyl urea analogues, which enabled the analysis of structure–activity relationships (SAR), with several derivatives having demonstrated up to a ∼30-fold increase in potency relative to frentizole.^104^

**Table 2:** Names and structures of benzothiazolyl urea inhibitors. Inhibitor names are shown as they appear in the original literature

Compound	IC_50_ (µM)	Assay type	Inhibition type	Structure	Ref.
Frentizole	200	ELISA based	Aβ-interaction	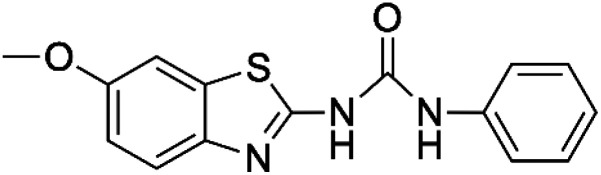	104
5h	6.46	ELISA based	Aβ-interaction	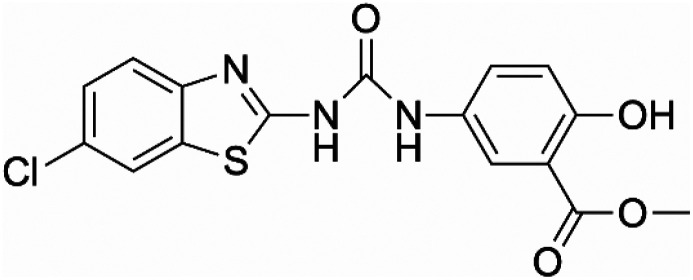	104
5l	6.56	ELISA based	Aβ-interaction	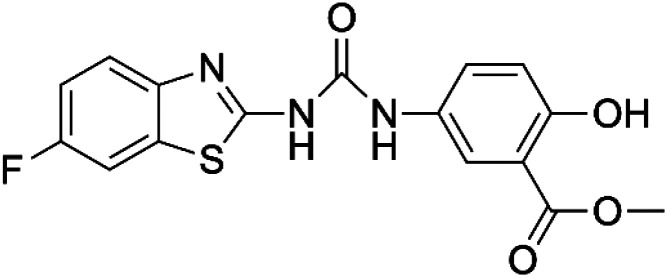	104
Compound 9	0.34	Recombinant protein	Enzymatic	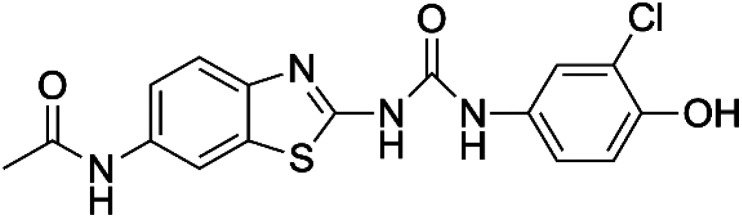	105
Compound 11	0.31	Recombinant protein	Enzymatic	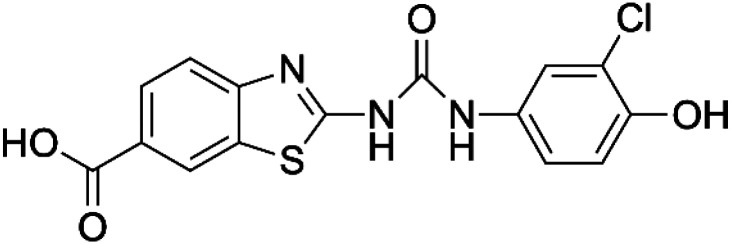	105
Compound 5	1.28	Recombinant protein	Enzymatic	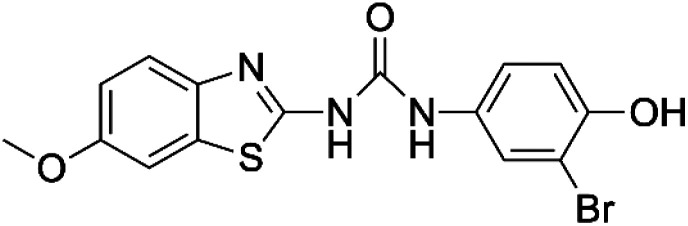	102
Compound 6	1.86	Recombinant protein	Enzymatic	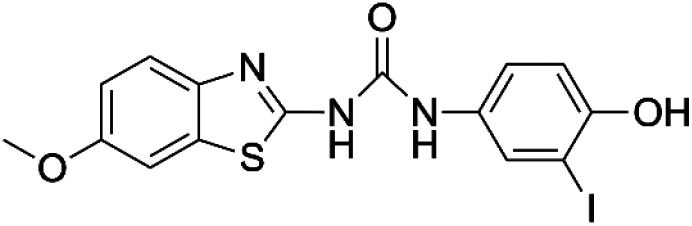	102

These SAR studies revealed the urea, rather than an amide moiety, to be essential for inhibition of 17β-HSD10–Aβ protein–protein interactions. The authors attributed this key pharmacophore element to the hydrogen-bond donor ability of the urea-NH groups. The addition of a *para*-hydroxyl group to the phenyl-urea ring yielded a hydroxyphenyl urea derivative with increased potency, whilst further modifications combining hydroxyl and methoxycarbonyl functionalities led to the development of the most potent compounds 5h and 5l (Table 2).^104^

A later study conducted by Hroch *et al.* (2016)^101^ showed a 4-phenolic moiety with chlorine in close proximity to be crucial for 17β-HSD10 inhibition, with a 3-halogen/4-hydroxyl substitution on the distal phenyl ring demonstrating potent inhibition. These findings provided a foundation for further optimisation and refinement of candidate compounds. Consequently, Aitken *et al.* (2019)^102^ synthesised and expanded upon this, thereby identifying compounds 5 and 6 (Table 2) as the most promising candidates featuring either a 3-bromo or 3-iodo in conjunction with a 4-hydroxyl substitution. Enzyme inhibition assays showed that both compounds act *via* reversible, mixed-type inhibition. This mechanism contrasts with the then “gold standard” 17β-HSD10 inhibitor, AG18051 (Table 3) (see below), which exerts inhibition *via* an irreversible covalent modification of the NAD^+^ cofactor.^90^ Thus, the benzothiazolyl ureas’ mixed reversible mode of inhibition is favourable as it implies greater selectivity and reduced off-target reactivity toward other NAD^+^-dependent short-chain dehydrogenase/reductase (SDR) family members.^102^

**Table 3:** Names and structures of pyrazole-derived inhibitors. Inhibitor names are shown as they appear in the original literature

Compound	IC_50_ (µM)	Assay type	Inhibition type	Structure	Ref.
AG18051	0.092	Recombinant protein	Enzymatic	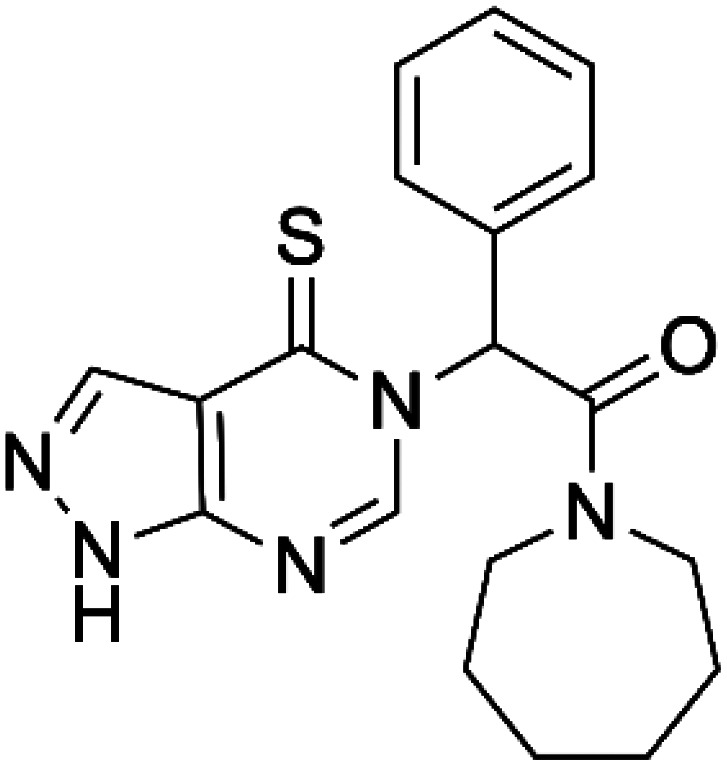	90
Compound 14b	0.74	ELISA based	Enzymatic	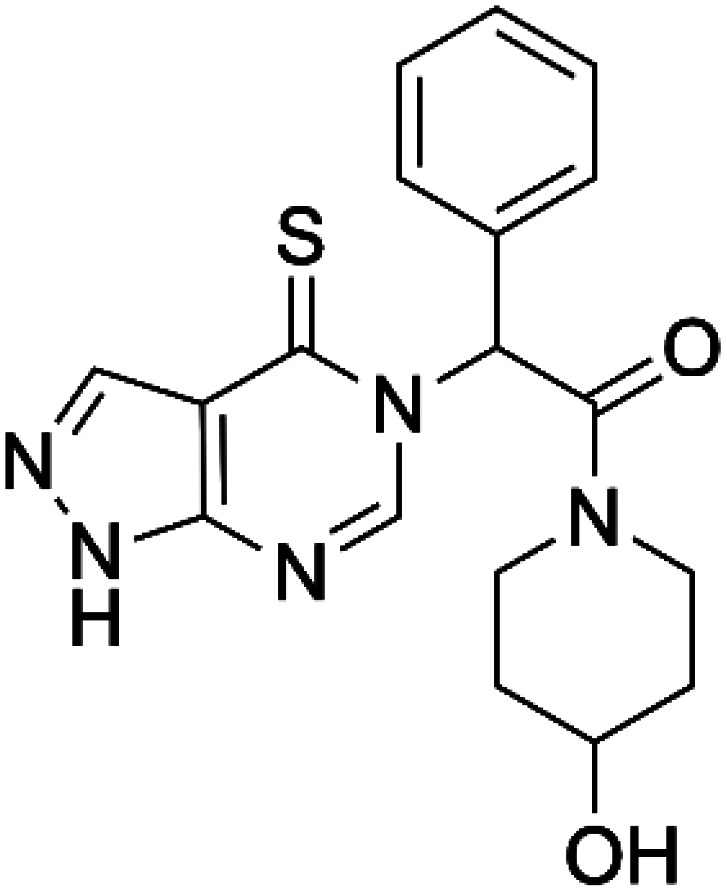	106

Recent studies have further probed the effects of substitutions on the benzothiazolyl core, specifically at the C6 position,^105^ where either a large hydrogen-bond acceptor or a small hydrogen bond donor modulates multiple electrostatic and steric interactions within the enzyme binding site. Subsequent optimisation led to the identification of submicromolar inhibitors, notably compounds 9 and 11, which have IC_50_ values of 0.34 µM and 0.31 µM, respectively (Table 2).^105^

Taken together, these findings show the evolution of benzothiazolyl urea entities from a known class of compounds, repurposed into a chemically distinct class of 17β-HSD10 inhibitors with significantly improved potency and selectivity.

## Pyrazole derivatives

For many years, the most potent and widely studied inhibitor of 17β-HSD10 was AG18051 (Table 3), with an IC_50_ of 92 nM.^90^ Structurally, AG18051 is 1-azepan-1-yl-2-phenyl-2-(4-thioxo-1,4-dihydro-pyrazolo[3.4-*d*]pyrimidin-5-yl)-ethanone, whereby its N_2_ atom forms a covalent adduct with the C4 carbon atom on the nicotinamide ring of the 17β-HSD10-bound NAD^+^ cofactor. This irreversible modification disrupts the 17β-HSD10-NAD^+^ holoenzyme, leading to suppressed activity. However, the covalent nature of this inhibition raises concerns regarding off-target reactivity and therapeutic viability.^90^

Molecular dynamic simulations provided further insight into the structural relationship between AG18051 and its binding site.^107^ These studies highlighted substantial conformational flexibility displayed by the azepane and benzene rings of AG18051, which correlates with the dynamic mobility of the 17β-HSD10 substrate-binding loop.^107^ This suggests that, in the absence of a ligand, the substrate-binding loop remains highly flexible, whereas AG18051 binding leads to stabilisation of the active site.^107^ In cell culture assays, using SH-SY5Y (human neuroblastoma) cells, AG18051 was shown to reduce ROS formation, protect against Aβ-induced cytotoxicity and prevent the Aβ-mediated decrease in estradiol.^108^ Such neuroprotective effects lend significant merit to pursue 17β-HSD10 as a therapeutic target for the treatment of AD.

Despite its potency and well-documented mechanism of action, the irreversible covalent binding mode raises concerns over off-target effects, specifically for other essential NAD^+^-dependent SDR enzymes, thus limiting translational applicability and precluding AG18051 as a viable candidate for AD therapy. AG18051, however, continues to serve as the standard reference compound for both *in vitro* and *in vivo* studies and remains a valuable benchmark control for the synthesis of novel 17β-HSD10 inhibitors.

Building upon the fused-pyrazole structure of AG18051, Morsy *et al.* (2022)^106^ replaced the original azepane ring with alternative nitrogen-containing heterocycles to create a series of ‘functionalised allopurinols’ with improved physiochemical properties and brain penetrance. Within this series, compound 14b (LD14b) (Table 3) had the original azepane ring replaced with a piperidinyl alcohol group, markedly increasing potency and optimisation scores. *In silico* modelling suggests that LD14b stabilises 17β-HSD10 by engaging Gly199, Thr203 and Leu22 within the Aβ-interface.^106^ In E2 rescue assays, LD14b presented submicromolar inhibition with an IC_50_ of 0.74 µM, in addition to protecting SH-SY5Y cells from Aβ-induced cytotoxicity and preventing Aβ-induced mitochondrial dysfunction and abnormal mitochondrial morphology in cortical neurons derived from 5XFAD mice.^106^ In light of these results, Daria *et al.* (2024)^109^ conducted detailed absorption, distribution, metabolism and excretion (ADME) studies to determine pharmacokinetic (PK) parameters for LD14b, showing good metabolic stability, with ∼70% of the parent compound detected in human liver S9 fractions after 90 minutes. Caco-2 permeability assays, demonstrated that LD14b exhibits intermediate intestinal drug absorption and BBB penetration capabilities. Furthermore, LD14b also showed moderate oral bioavailability with a 3–5 hour half-life and large volume of distribution, suggesting broad tissue exposure.^109^

## Steroidal derivatives

The demonstrated ability of 17β-HSD10 to metabolise steroidal substrates^34^ suggests that its active site could accommodate steroidal scaffolds, making steroidal derivatives a promising candidate for inhibitor development. As such, a variety of compounds were predicted to interact with the 17β-HSD10 binding pocket, and several steroid-based inhibitors were subsequently synthesised and evaluated for their ability to modulate enzymatic activity (Table 4).^43^

**Table 4:** Names and structures of steroidal derived inhibitors. Inhibitor names are shown as they appear in the original literature

Compound	IC_50_ (µM)	Assay type	Inhibition type	Structure	Ref.
RM-532-46	0.55	Cell based	Enzymatic	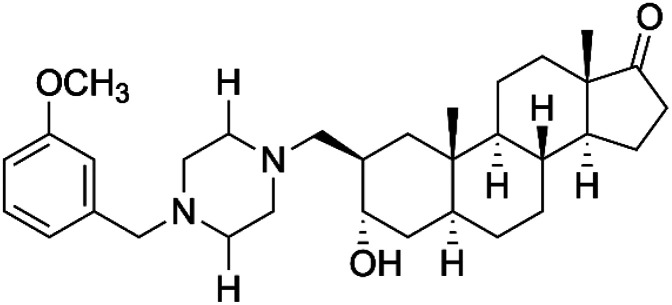	43
D-3,7	0.14	Cell based	Enzymatic	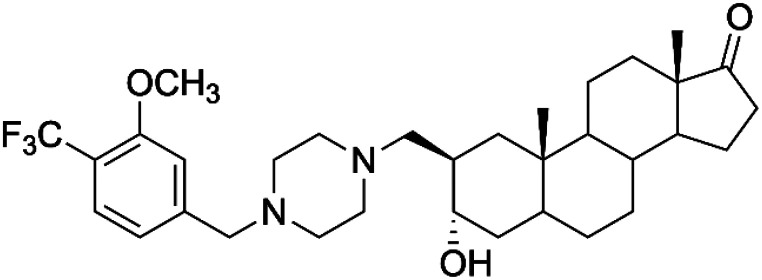	113
Compound 23	5.59	Recombinant protein	Enzymatic	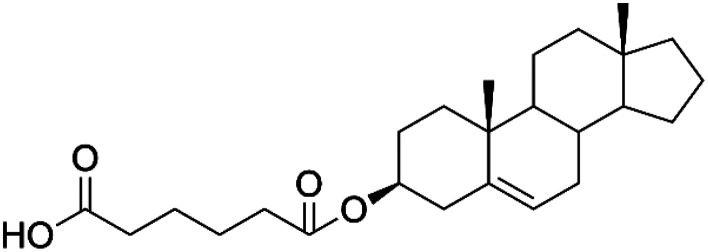	91

Among the candidates, a lead compound RM-532-46 (Table 4) showed promise, with an IC_50_ of 0.55 µM.^43^ Despite its potency, RM532-46 was deemed unsuitable due to being derived from DHT, a compound known to inhibit 17β-HSD3. This enzyme catalyses the conversion of androstenedione to testosterone,^42,110^ a key step in androgen biosynthesis. As prostate cancer is driven by androgen receptor signalling,^111^ inhibition of 17β-HSD3 poses as a potential therapeutic strategy to suppress androgen production. Consequently, this cross-reactivity suggests that RM-532-46 would likely be more appropriate as a drug targeting prostate cancer rather than a selective drug targeting AD.^43^

From the structure of RM-532-36, Boutin *et al.* (2018)^112^ synthesised a series of 15 analogues and evaluated inhibitory effects against two natural steroidal substrates, AlloP and E2. The results revealed substrate-dependent differences in inhibitory potency, where compound 5 was the most effective when assayed with AlloP and less so with E2, yielding IC_50_ values of 235 µM and 610 µM, respectively. As predicted, compound 5 also demonstrated the ability to cross the BBB, with a BBB penetration score of −2.61. In contrast, compound 8 showed the highest potency with the E2 substrate, with an IC_50_ of 300 µM, but exhibited minimal inhibition against AlloP, suggesting that certain structural features select for particular substrates.^112^

Further optimisation of the same RM-532-46 compound introduced different modifications to its core scaffold, including D-ring, side chain and dual hybrid modifications.^113^ These investigations yielded a D-ring-modified amine derivative, known as D-3,7 (Table 4), which demonstrated excellent potency with an IC_50_ value of 0.14 µM.^113^ Additional selectivity testing also indicated that D-3,7 had overcome the initial lack of specificity presented by RM-532-46^113^

In contrast, other derivatives, such as compound 23 (Table 4), demonstrated more moderate inhibitory activity with an IC_50_ of 5.59 µM,^91^ highlighting the variability in potency associated with structural modifications within steroidal derivatives.

## Repurposed and other compounds

With a surge in drug repositioning, several clinically established compounds have been repurposed for the treatment of additional diseases.^114^ One such compound is risperidone (Table 5), an FDA-approved drug for the treatment of schizophrenia and bipolar disorder.^115,116^ In a chemical genomic screen using a T7 bacteriophage display library, risperidone was identified as a potential 17β-HSD10 inhibitor. Enzyme assays demonstrated that 17β-HSD10 activity with E2 and acetoacetyl-CoA substrates resulted in oxidation of E2 and reduction of acetoacetyl-CoA at rates of 1.0 µM min^−1^ and 3.3 µM min^−1^, respectively.^117^ Moreover, *in silico* computational docking experiments ranked risperidone among the highest-scoring ligands for 17β-HSD10 and predicted the compound to act as a competitive inhibitor;^117^ however, empirical pharmacodynamic parameters and inhibitor specificity are yet to be determined, with further biochemical and structural validation required.

**Table 5:** Names and structures of novel and repurposed drugs as 17β-HSD10 inhibitors. Inhibitor names are shown as they appear in the original literature

Compound	IC_50_ (µM)	Assay type	Inhibition type	Structure	Ref.
Risperidone	—	Recombinant protein	Enzyamtic	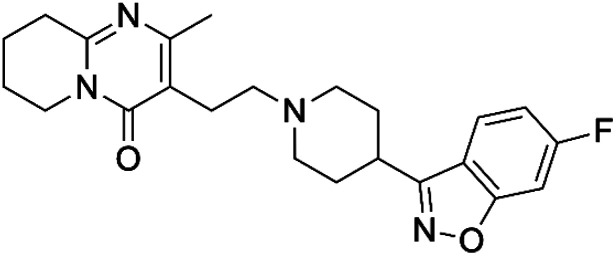	117
BCC0100281	25.1	Recombinant protein	Enzymatic	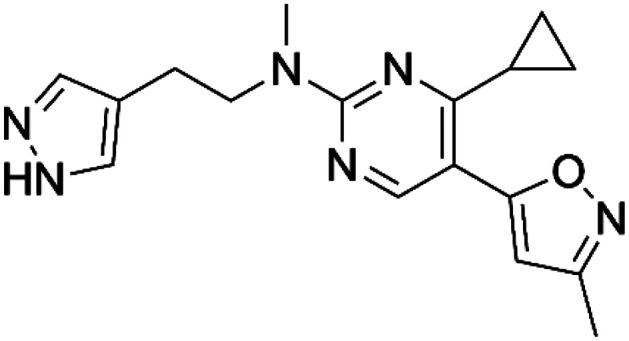	120
ESC1002755	0.019	Recombinant protein	Enzymatic	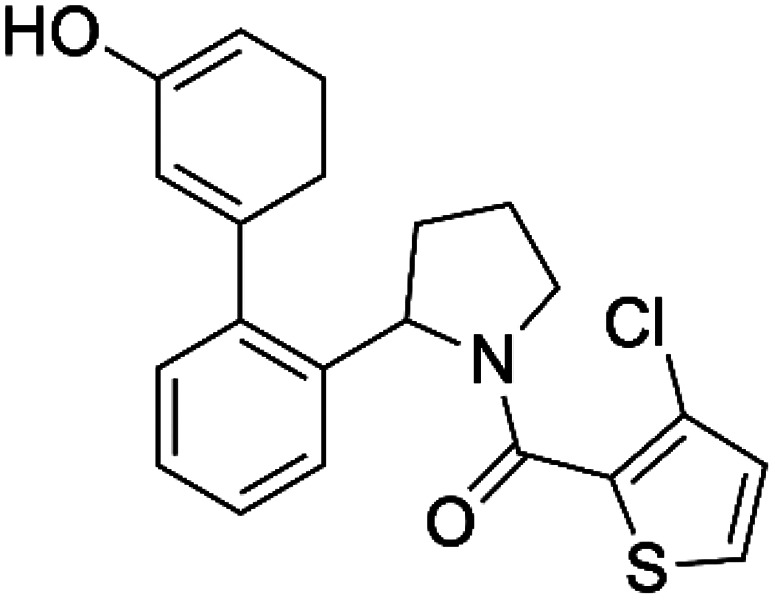	97
ESC1002576	0.25	Recombinant protein	Enzymatic	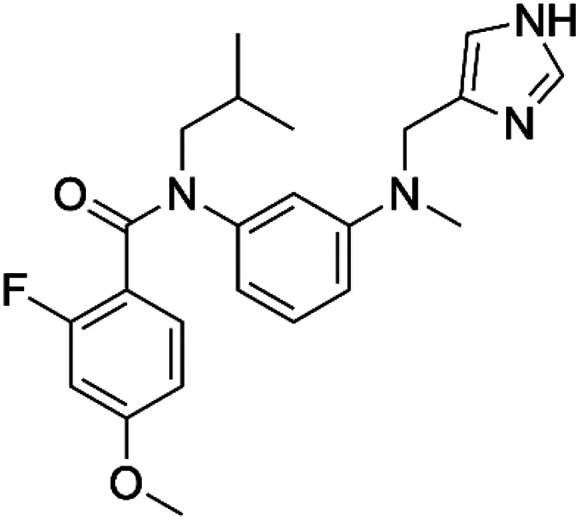	97

In 2017, Aitken *et al.* conducted a pilot high-throughput screening (HTS) of 6759 compounds, which identified 16 low-micromolar inhibitors of 17β-HSD10. Physiochemical profiling revealed that all hits exhibited favourable drug-like properties, establishing a strong foundation for subsequent SAR optimisation and *in vivo* validation, and thus enabling the development of 17β-HSD10-targeted therapeutics.^118^ Building upon this work, Dow *et al.* (2025)^119^ selected BCC0100281 (Table 5) for further investigation, in which cell-based assays displayed marked cytotoxicity in SH-SY5Y neuroblastoma cells, precluding further use in Aβ-induced cytotoxicity.^119^ Notably, this cytotoxic effect was not observed across a broader panel of cancer and non-cancer cell lines, highlighting a degree of cell-specific vulnerability. Further analysis by differential scanning fluorimetry (DSF) revealed a decrease in 17β-HSD10 thermal stability when assayed with BCC0100281, consistent with a non-canonical binding mode.^119^ Derivatives targeting modifications of the central pyrimidine scaffold were subsequently synthesised and found to exhibit protective effects against Aβ-induced cytotoxicity.^119^ These compounds, however, displayed an inverse dose–response relationship, indicating both mechanistic complexity and room for further optimisation. While BCC0100281 is unlikely to advance as a therapeutic lead, it nonetheless provides a valuable chemical starting point for the continued development of next-generation 17β-HSD10 inhibitors.

**Fig. 6:**
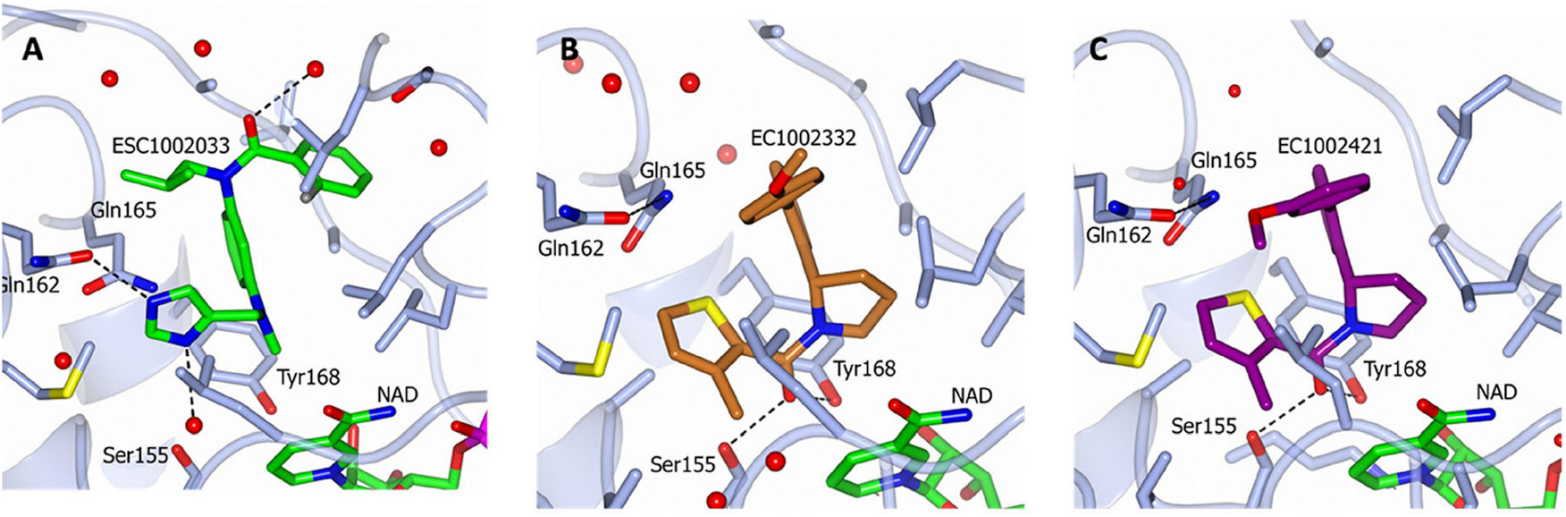
Interactions between 17β-HSD10 with specific novel compounds (A) ESC1002033, (B) ESC10023332 and (C) ESC1002421 sitting at a novel allosteric site. Figure taken from ref. 97, L. Aitken *et al.*, *ACS Chem. Biol.*, 2025, **20**, DOI: https://doi.org/10.1021/acschembio.5c00110. Published by the American Chemical Society under a CC BY 4.0 licence.

In 2025, Aitken *et al.* published the first industrial-scale HTS of 350 000 drug-like molecules, generating several lead series for the potent inhibition of 17β-HSD10. From the screening, two distinct compound series emerged for future development, a singleton hit series, ESC1002755 (Table 5) and a chemically related hit series referred to by the authors as cluster 6. From the cluster 6 series, ESC1002576 (Table 5) was selected as a lead compound. Although ESC1002576 did not exhibit the highest potency within cluster 6 (IC_50_ = 0.25 µM), it possessed the most favourable selection based on its overall potency and physiochemical properties.^97^ESC1002755 (Table 5) yielded the highest potency against 17β-HSD10, with an IC_50_ of 19 nM in enzyme activity assays and an EC50 of 28 nM during overexpression of the canonical form in HEK293.^97^ Additionally, binding site analysis supported by co-crystallography of related compounds identified a novel allosteric site on 17β-HSD10 (Fig. 6). The novel allosteric site is bordered by Gln162, Gln165, Ser155 and Tyr168 residues, with the allosteric site and residues sitting adjacent to, but not overlapping the NAD^+^ cofactor binding site, explaining the non-competitive and competitive inhibition of NADH and acetoacetyl-CoA respectively. This allosteric mode of action distinguishes ESC1002755 from classic 17β-HSD10 active site inhibitors such has AG18051.^97^ Beyond its potency, ESC1002755 showed optimal selectivity and safety profiles in several cell-based assays, with minimal cell cytotoxicity.^97^ Moreover, screening of ESC1002755 against other SDR enzymes showed high selectivity for 17β-HSD10, while *in vitro* ADME assessments yielded favourable pharmacokinetic profiles.^97^

## Future perspectives

Significant strides have been made in the chemical design and optimisation of 17β-HSD10 inhibitors, which have resulted in the identification and synthesis of an array of compounds that span multiple chemical classes, with several compounds achieving submicromolar potency.^97,105,113^ Despite this progress, relatively little work has focused on their functional evaluation in physiologically relevant neuronal systems. Cellular studies, however, are moving in the right direction, with experiments being conducted using undifferentiated or non-neuronal cell models including HEK293 and SH-SY5Y neuroblastoma cell lines, as well as primary cortical neurons from AD mouse models.^15,20,21,94,97,106,109,119^ While these models have provided useful initial screening platforms, they still do not capture the complexity of the true *in vivo* microenvironment; more recent investigations, however, have been developing this capability. Houfková *et al.* (2025),^94^ for example, developed stable monoclonal HEK293 cell lines overexpressing HSD10, a catalytically inactive mutant, and an AD-associated mutation, APPSwe/Ind variant, thus allowing direct observation of the enzymatic and non-enzymatic contributions of HSD10 to cellular pathology. Critically, the study demonstrated that HSD10 overexpression alone is enough to induce mitochondrial dysfunction and cytotoxicity, independent of Aβ. This cytotoxicity was dependent upon HSD10 enzymatic activity, as the catalytically inactive HSD10 mutant did not present the phenotype. Importantly, cytotoxicity was only observed under glucose-deprived conditions,^94^ where cells are forced to rely upon mitochondrial oxidative phosphorylation, highlighting the critical role of metabolic context in modulating HSD10-driven toxicity.

Metabolic profiling further revealed that HSD10 overexpression led to broad suppression of tricarboxylic acid cycle activity and impaired β-oxidation. In contrast, the APPSwe/Ind cells exhibited increased glucose utilisation and upregulated β-oxidation, reflecting a compensatory mechanism to Aβ-induced mitochondrial stress.^94^

Despite emerging insights into 17β-HSD10 function, the efficacy and safety of these inhibitors in differentiated neuronal cell types, including astrocytes, oligodendrocytes and microglia, remain largely unknown, despite evidence linking 17β-HSD10 and its associated metabolic pathways to stress modulation in astrocytes and differentiation processes in oligodendrocytes.^16^ These findings emphasise the need to extend pharmacological assessments beyond the generic immortalised models to achieve a more accurate representation of physiological context. Indeed, the rise of mass spectrometry capabilities with these new potent inhibitor compounds will allow it to be possible to identify the metabolic changes caused by 17β-HSD10 activity in the living brain. As such, it is crucial that drug development pipelines for 17β-HSD10 inhibitors incorporate cellular differentiation assays and human induced pluripotent neuronal stem cell models. These approaches will shed light on how candidate compounds interact with distinct neural populations and will also provide mechanistic insights into their therapeutic action and neurotoxicity in disease-relevant contexts.

## Conflicts of interest

There are no conflicts to declare.

## Data Availability

No primary research results, software or code have been included, and no new data were generated or analysed as part of this review.
